# Advanced Functionalized CeO_2_/Al_2_O_3_ Nanocomposite Sensor for Determination of Opioid Medication Tramadol Hydrochloride in Pharmaceutical Formulations

**DOI:** 10.3390/nano12081373

**Published:** 2022-04-16

**Authors:** Seham S. Alterary, Maha F. El-Tohamy

**Affiliations:** Department of Chemistry, College of Science, King Saud University, P.O. Box 50013, Riyadh 11523, Saudi Arabia; moraby@ksu.edu.sa

**Keywords:** tramadol hydrochloride, CeO_2_/Al_2_O_3_ nanocomposite, modified sensor, commercial products

## Abstract

Background: The exceptional characteristics of cerium oxide (CeO_2_) and aluminum oxide (Al_2_O_3_) nanoscales have inspired significant attention to those nanocomposites as possible electroactive resources for applications of sensing and biosensing. Methods: In this research, an innovative new factionalized CeO_2_/Al_2_O_3_ nanocomposite membrane sensor was presented to assess tramadol hydrochloride (TRD) in marketable products. Results: Tramadol-phosphomolybdate (TRD-PM) was formed by mixing tramadol hydrochloride and phosphomolybdic acid (PMA) in the attendance of polymeric matrix and *o*-nitrophenyloctyl ether solvent mediator. With 1.0 × 10^−10^–1.0 × 10^−2^ mol L^−1^ as a range of linearity and E_mV_ = (57.567 ± 0.2) log [TRD] + 676.29 as a regression equation, the functionalized sensor using TRD-PM-CeO_2_/Al_2_O_3_ nanocomposite showed great selectivity and sensitivity for the discriminating and measurement of TRD. Using the regression equation E_mV_ = (52.143 ± 0.4) log [TRD] + 431.45, the unmodified coated wire sensor of TRD-PM, on the other hand, showed a Nernstian response between 1.0 × 10^−6^ and 1.0 × 10^−2^ mol L^−1^, Using the methodology’s specified guidelines, the proposed improved potentiometric system was validated against several criteria. Conclusion: The suggested method is suitable for the determination of TRD in its products.

## 1. Introduction

The evolution of modified sensing and biosensing probes has been aided by advances in nanoscience technologies and nanomaterial engineering, which have opened up new fields in scientific inquiry. The recent research has concentrated on the creation of nanocomposites rather than single nanoparticles. Because of their interfacial interactions, these nanocomposites frequently have various nanoscale domains, which produce synergistic effects [1]. Chemical resistance, high conductivity, biocompatibility, and flexibility are just a few of the advantages that nanocomposites have over traditional polymers [2]. Recent advancements in scientific domains such as Recent advancements in scientific domains, such as industry [3], biology [4], and material science [5] need the creation of innovative sensing knowledge that associates low power consumption, compactness, and high tangible sensitivity.

Nanocomposite is a high-activity nanostructure that offers a wide range of engineering and combining options. Their potential is so great that can be effectively utilized in a diversity of sensing and biosensing approaches [6], due to their expanding requirement and speedy inquiry to be in the fabrication of sensors; indeed, they have emerged as viable options for addressing the disadvantages of micro composites. Additionally, these materials have exceptional structures and optical properties which are not seen in traditional types [7]. Furthermore, nanocomposite production is regarded as a critical step in the formation of a huge number of electronics [8,9], systems of drug targeting [10,11], medicinal, and immunosensing probes [12].

Metal oxides, such as aluminum oxide (Al_2_O_3_), copper oxide (CuO), nickel oxide (NiO), and others, are currently the subject of various investigations. Furthermore, the use of cerium oxide (CeO_2_) in different fields such as antibacterial agents [13], catalysis [14], food packaging [15], sensors [16], agriculture [17], and medicine [18] has received greater attention. The development of aluminium oxide nanoparticles (Al_2_O_3_NPs) with a high specific surface area and outstanding optical, catalytic, and thermal stability properties for progressive engineering and industrial applications has recently gained attention [19,20,21,22]. Few studies suggested the use of CeO_2_/Al_2_O_3_ nanocomposite in sensing applications such as Naik et al. [23] described a successful synthesis of CeO_2_/Al_2_O_3_ nanocomposite using the solution combustion method. The gas sensing characteristics of synthesized CeO_2_/Al_2_O_3_ nanocomposite have been studied for NO_2_ under various temperature and concentrations conditions; moreover, Krishnan et al. [24] studied the cathodic potential of the Ni-P sensor for analysis of alkaline water by the catalytic activity of CeO_2_-Al_2_O_3_ nanocomposite. Another study was developed by Avramova et al. [25], which employed the chemical deposition of CeO_2_-Al_2_O_3_ nanocomposite on stainless steel electrodes. Different microscopic and spectroscopic procedures, including scanning electron microscope (SEM) and X-ray photoelectron spectroscopy (XPS), were employed to examine the thickness of the coating layer and the chemical constituents with respect to the loading ceria. In recent studies, sol-gel, microwave-assisted, electrochemical deposition methods have all been applied for the synthesis of CeO_2_NPs and Al_2_O_3_NPs [26,27,28,29,30,31].

The potentiometric systems are more dependable and cost-effective for various biomedical aspects [32,33,34,35]. These approaches are also fast with respect to the duration of analysis [36]. Several biosensing electrodes have been functionalized with metallic oxides to improve their sensitivity and detection limitations [37,38]. Potentiometric sensors usually comprise membranes developed from polyvinyl chloride (PVC) with high molecular weight, plasticizers such as dioctyl sebacate (DOS), dioctyl phthalate (DOP), dibutyl sebacate (DBS), dibutyl phthalate (DBP), and o-nitrophenyl octyl ether (o-NPOE) as a solvent mediator, and dibutyl These membranes can also be made of lipophilic ions or molecules, which operate as active materials to induce specific analyte interactions in the membrane locations, allowing for the pre-detection of the sensor selectivity [39].

Potentiometric wire-based sensors are often made of metal wire of a high conductivity such as copper, iron, silver, aluminum, and platinum. The selective membrane’s active spots are coated on a polymeric cocktail that is applied to the metal wire as a substrate. Phosphomolybdic acid (PMA) is a yellow-green powder that has the ability to dissolve in water and alcohol. When combined with an appropriate ion-pair complex, it can also catalyze the process of ion-exchange process via the hydrophobic membranes’ interfaces [40].

**Figure 1:**
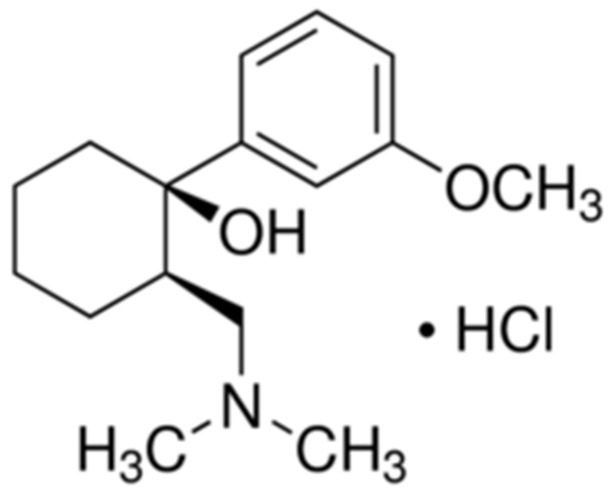
Chemical structure of tramadol hydrochloride.

Tramadol hydrochloride is a pain relief compound that is utilized to treat pain in a moderate-to-severe sense (Figure 1).

Tramadol’s extended-release pills and capsules are only prescribed for those who are likely to require pain relief 24 h a day; it belongs to the opiate (narcotic) analgesics class of drugs [41].

Several analytical approaches, such as spectroscopic [42], chromatographic separation [43,44,45], and electrochemical methods [46,47], have previously been used to assess and quantify TRD. Although these previously published methods had good sensitivity and selectivity for TRD detection, the reminder had some drawbacks, such as requiring a long analytical time, a high level of operating skills, and the use of huge volumes of solvents.

The goal of this research was to develop a modified metal oxide (CeO_2_/Al_2_O_3_) nanocomposite coated wire sensor that could detect TRD in commercial items with high sensitivity and selectivity. To improve the sensitivity and selectivity of the potentiometric modified sensor, a new technique based on utilizing the exceptional physical, chemical, optical, and conductive features of the chosen metal oxides has been proposed. The integration of CeO_2_/Al_2_O_3_ in a polymeric matrix will have an impact on the suggested sensor’s sensitivity and selectivity for the selected drug. Method validation follows ICH criteria [48] to confirm the indicated method’s analytical appropriateness. In addition, a comparison was made between the CeO_2_/Al_2_O_3_ nanocomposite coated membrane sensor proposed and the normally built kind.

## 2. Materials and Methods

### 2.1. Chemicals and Reagents

Amyria Pharmaceuticals (Alexandria, Egypt) provided pure grade opioid pain medicine tramadol hydrochloride and Tramadol^®^ capsules (50 mg/capsule). Sigma Aldrich, Hamburg, Germany, provided ortho-nitrophenyloctyl ether (o-NPOE), acetone 99.9%, methanol 99.9%, ethanol 99.9%, tetrahydrofuran (THF) 97.0%, hydrochloric acid 37%, and high molecular weight PVC, among other analytical chemicals and solvents. BDH yielded 99.0% cerium nitrate, 99.9% aluminum nitrate nonahydrate, phosphomolybdic acid (PMA), and 99.9% sodium hydroxide (Poole, UK).

### 2.2. Instruments

The designed potentiometric system included a manufactured standard tramadol hydrochloride-phosphomolybdate (TRD-PM) or functionalized TRD-PM-CeO_2_/Al_2_O_3_ nanocomposite coated wire sensor, as well as a reference silver/silver chloride (Ag/AgCl) sensor (JEOL Ltd., Tokyo, Japan). Furthermore, the presence of Ce, Al, and O elements in the synthesized nanomaterials was detected using Energy-Dispersive X-ray Spectroscopy (EDX) analysis combined with EDX-8100 (Shimadzu, Kyoto, Japan). Different spectroscopic and microscopic methods were applied to characterize the synthesized metal oxide nanoparticles and nanocomposite, including UV-2450 spectrophotometer (Shimadzu Corporation, Kyoto, Japan), the Fourier-Transform Infrared spectroscopy (FT-IR) Spectrum BX spectrometer (Shimadzu Corporation, Kyoto, Japan), and the UV-2450 spectrophotometer (Shimadzu Corporation, Kyoto, Japan (PerkinElmer, Waltham, Massachusetts, United States). Shimadzu XRD-6000 diffractometer (Shimadzu, Kyoto, Japan), JSM-7610F scanning, and transmission electron microscopes (SEM-JEOL and TEM-JEM-2100F Ltd., Tokyo, Japan) were used for microscopic investigations. Furthermore, Energy-Dispersive X-ray Spectroscopy (EDX) analysis utilizing the (EDX-8100, Shimadzu, Kyoto, Japan) was utilized for elemental analysis.

### 2.3. Preparation of TRD-PM Electroactive Material

The electroactive complex TRD-PM was made by combining similar volumes (50 mL) of aqueous TRD and PMA solution with an equimolar concentration (1.0 × 10^−2^ mol L^−1^) of TRD. A greenish TRD-PM precipitate was formed. The precipitate was cleaned with Milli Q water and kept to dry overnight after being filtered with Whatman filter paper No. 41.

### 2.4. Synthesis of CeO_2_ and Al_2_O_3_ Nanoparticles

CeO_2_NPs were synthesized by the preparation of 50 mL of 0.5 mol L^−1^ of cerium nitrate hexahydrate in Milli Q water as a precursor solution. With constant stirring and at ambient temperature, 2.0 mol L^−1^ of NaOH was dripped slowly. The addition was performed within 30 min. The mixture was centrifuged at 2500 rpm for 10 min. The precipitate was collected using Whatman filter paper No. 1, then rinsed thoroughly with Milli Q water. The formed CeO_2_NPs were dried for 6 h at 100 °C. To evaporate the water, the formed nanoparticles were calcinated in a furnace oven at 600 °C for 4 h.

A sol-gel method was used to synthesize Al_2_O_3_NPs by mixing 50 mL of aluminum nitrate (2.0 mol L^−1^) with 20 mL of citric acid and the mixture was stirred at 250 rpm for 30 min. The prepared solution was heated under magnetic stirring at 60 °C for a further 30 min until the formation of while gel. The formed gel was heated to 80 °C under continuous stirring until a transparent gel was formed. The resulting nanoparticles were filtered after 10 min centrifugation at 2500 rpm. The collected Al_2_O_3_NPs were washed three times with Milli-Q water, oven-dried at 90 °C for 12 h, and thereafter sintered for 4 h at 600 °C.

### 2.5. Preparation of Polymeric TRD-PM-CeO_2_/Al_2_O_3_ Nanocomposite

The polymeric functionalized solution of CeO_2_/Al_2_O_3_ nanocomposite was formed by suspending approximately 5 mg of each pre-synthesized CeO_2_ and Al_2_O_3_ nanoparticles with 10 mg TRD-PM complex, 190 mg of polymeric materials (PVC), and 0.35 mL of o-nitrophenyloctyl ether in 7 mL of THF. Under constant magnetic stirring to produce a polymeric solution of TRD-PM-CeO_2_/Al_2_O_3_ nanocomposite. Then it was utilized to functionalize the surface of the designed modified TRD-PM-CeO_2_/Al_2_O_3_ nanocomposite sensor.

### 2.6. Preparation of Standard TRD Solution

A TRD standard solution (0.1 mol L^−1^) was made by adding 2.998 g of TRD authentic powder to deionized water (100 mL). The analytical testing samples were diluted in the range of 1.0 × 1.0^−10^–1.0 × 10^−2^ mol L^−1^ using the same solvent.

### 2.7. Sensor Design and Membrane Composition

**Figure 2:**
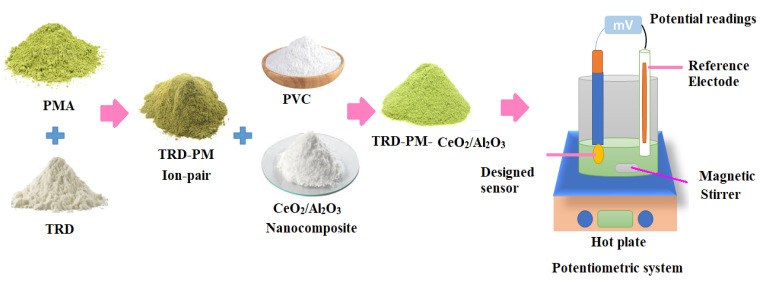
Preparation of TRD-PM-CeO_2_/Al_2_O_3_ electroactive complex and the potentiometric system employed for TRD determination.

A Typical (TRD-PM) sensor was designed using mixing-electroactive substances (TRD-PM, 10 mg), (PVC, 190 mg), and o-NPOE, 0.35 mL plasticizer in 5 mL of THF. The resulting cocktail was placed into a rounded dish and allowed to gently evaporate at room temperature. Deionized water, followed by acetone, was used to polish and clean the aluminum wire’s tip. The wire’s tip had been cleaned. The cleaned tip of the wire was submerged in the polymeric membrane solution (TRD-PM) many times until a coated membrane formed on its surface. Additional clean Al wire was dipped three times in the polymeric solution of CeO_2_/Al_2_O nanocomposite to generate a thin layer membrane on its surface for the modified sensor. After allowing the sensor to dry, it was dipped multiple times in the aforementioned polymeric (TRD-PM) solution until it formed a homogenous covered membrane. The cell assembly: Al wire/coated membrane/test solution/Ag/AgCl reference electrode was used in both constructed sensors. The potentiometric system and the functionalized TRD-PM-CeO_2_/Al_2_O_3_ nanocomposite sensor were illustrated, as shown in Figure 2.

### 2.8. Calibration Graph

The potential readings (mV) of (TRD-PM) and (TRD-PM-CeO_2_/Al_2_O_3_) nanocomposite sensors were measured and graphed versus -logarithm TRD concentrations (mol L^−1^). The linearity was estimated separately using TRD standard solutions (50 mL) in the concentration range 1.0 × 10^−10^–1.0 × 10^−2^ mol L^−1^ and the constructed functional TRD-PM or TRD-PM-CeO_2_/Al_2_O_3_ sensors were used and the applied reference one was (Ag/AgCl) electrode. The membrane surface should be cleaned using Milli-Q water and dried with soft paper before each measurement.

### 2.9. Optimization of Analytical Conditions

The pH of the examined solutions can have a substantial impact on the potential response of the coated wire sensors that have been designed. The suitable pH range using TRD (1.0 × 10^−5^ mol L^−1^) solution was measured using TRD-PM and modified TRD-PM-CeO_2_/Al_2_O_3_ nanocomposite sensors were measured. The acidity and alkalinity of the test sample were adjusted using 0.1 mol L^−1^ of hydrochloric acid and sodium hydroxide. The pH graphs were created by plotting the change in potential vs. pH.

Selectivity of the studied TRD sensors was monitored by exploiting a separate solution approach [49]. The selectivity coefficient of each sensor for TRD and various foreign substances and additives such cations (Na^+^, K^+^, Ag^+^, Mg^2+^, Ca^2+^, Zn^2+^, and Fe^3+^), sugars (lactose, Fructose, and starch), amino acids (histidine, glycine, lysine, and tryptophan) have been tested. The selectivity of the suggested sensors was measured using 1.0 × 10^−3^ mol L^−1^ solution of TRD and interferent species, separately. The tolerable value (K_pot_TRD^+^) was estimated from the previously reported equation [49].

The response time was determined by recording the dynamic sensors response of the investigated TRD solution, using a TRD working concentration range.

### 2.10. Quantification of Tramadol Hydrochloride^®^ Capsules

The content of 10 tramadol hydrochloride^®^ capsules (50 mg/capsules) was mixed well and weighed. A precise amount (0.2998 g in 50 mL Milli-Q water) was centrifuged (5 min at 1500 rpm), and the co-formulated components were removed by filtering. Deionized water was used to complete the clear solution to be 100 mL. The same solvent was used to dilute the resulting TRD solution (1.0 × 10^−2^ mol L^−1^) to prepare the working samples in the range of 1.0 × 10^−5^–1.0 × 10^−2^ and 1.0 × 10^−10^–1.0 × 10^−2^ mol L^−1^. The investigated drug was quantified in commercial capsules using the developed TRD-PM and functionalized TRD-PM-CeO_2_/Al_2_O_3_ nanocomposite sensors independently.

## 3. Results and Discussion

### 3.1. Characterization of CeO_2_/Al_2_O_3_ Nanocomposite

**Figure 3:**
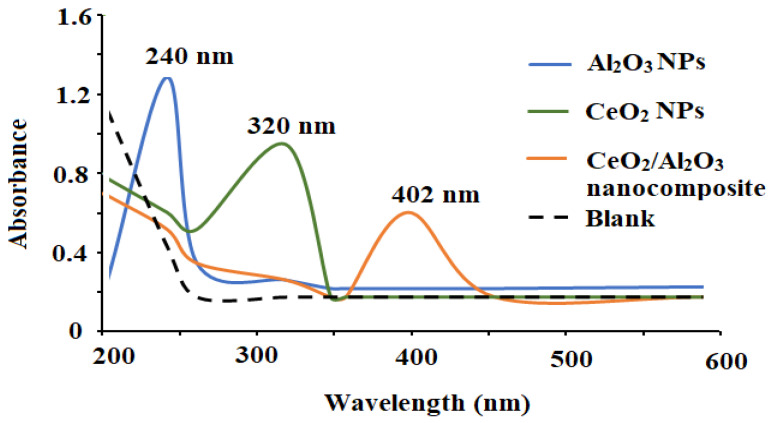
The optical spectra of the synthesized CeO_2_NPs, Al_2_O_3_NPs, and CeO_2_/Al_2_O_3_ nanocomposite measured at wavelength 200–600 nm.

Various spectroscopic investigations such as XRD, UV-Vis, FT-IR, and EDX were performed to characterize and confirm the formation of the synthesized CeO_2_/Al_2_O_3_ nanocomposite. The UV-Vis analysis is one of the top appropriate and helpful ways for principal validation of the form, size, and stability of designed nanoparticles in their aqueous suspensions. The optical absorbance spectra of CeO_2_, Al_2_O_3_, and CeO_2_/Al_2_O_3_ nanocomposite were measured at 200–600 nm and exhibited three large absorption peaks at 320, 240, and 402 nm for CeO_2_NPs, Al_2_O_3_NPs, and CeO_2_/Al_2_O_3_ nanocomposite, respectively (Figure 3).

**Figure 4:**
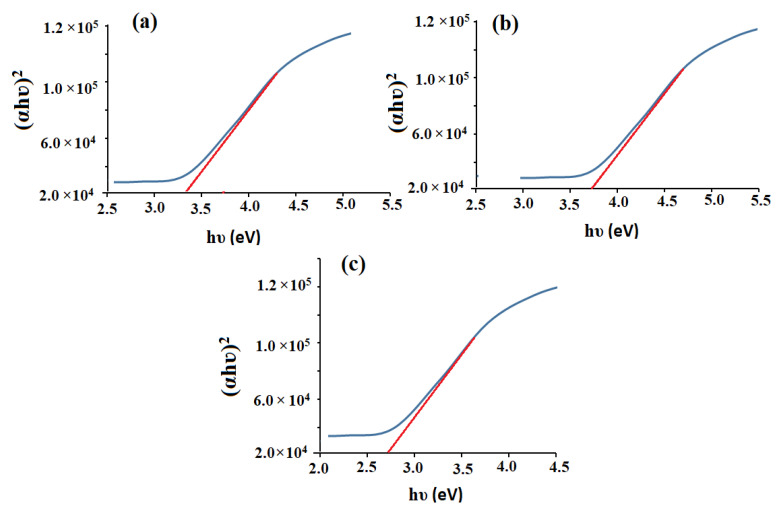
Bandgaps of the pre-synthesized (**a**) CeO_2_NPs, (**b**) Al_2_O_3_NPs, and (**c**) CeO_2_/Al_2_O_3_NPs.

The produced bandgaps of the metal oxide nanoparticles were determined obeying the formula:

Eg = hυ = hc/λ (1)

where h, c, and λ are Planck’s constant, light velocity, and absorption wavelength, respectively. On applying the Tauc plot function, the estimated optical bandgaps energy of CeO_2_NPs, Al_2_O_3_NPs, and CeO_2_/Al_2_O_3_ nanocomposite were found to be 3.36, 3.68, and 2.70 eV, respectively [50,51] (Figure 4a–c). Because of redshift, the bandgap energy difference between CeO_2_NPs and CeO_2_/Al_2_O_3_ nanocomposite was 0.66 eV, while the gap energy difference between Al_2_O_3_NPs and CeO_2_/Al_2_O_3_ nanocomposite was 0.97 eV. The decrease in bandgap energy in CeO_2_/Al_2_O_3_ nanocomposite improves the electron active sites to the entire movement on the Al_2_O_3_ surface, and their interaction might speed up the oxidation process. The surface plasmon resonance improves radiation penetration, creates scattering probability, and supplies the surface with a reduction form. These activities entail the formation of holes and the separation of electrons on the surface, which improves the oxidation process. Furthermore, changes in the dielectric matrix have been shown to influence the position of the SPR’s absorbance peak. The effective dielectric function of the matrix is known to have a direct relationship with the refractive index. A rise in the refractive index is promoted by the crystallization of CeO_2_NPs (*n* = 2.20) to (*n* = 3.54), and AL_2_O_3_NPs (*n* = 1.33) to (*n* = 1.76). This adjustment causes a red shift in the absorbance peak due to an increase in the dielectric function values [52].

**Figure 5:**
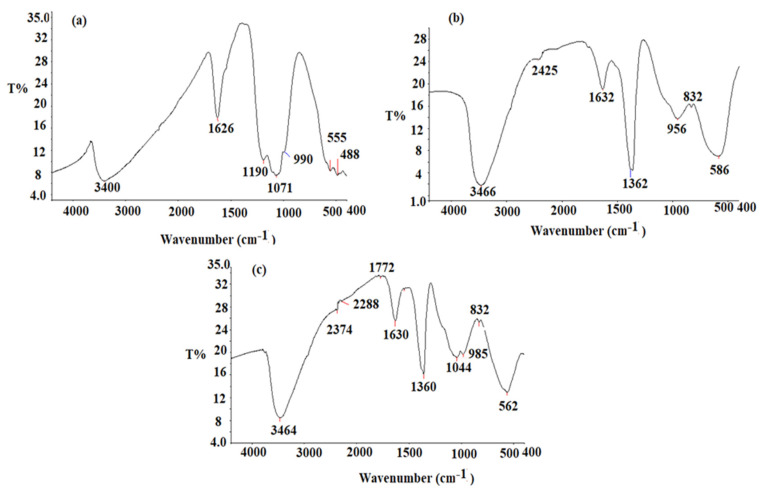
FT-IR spectrum of pre-synthesized (**a**) CeO_2_NPs, (**b**) Al_2_O_3_NPs, (**c**) CeO_2_/Al_2_O_3_ nanocomposite measured at wavenumber 400–4000 cm^−1^.

The pre-synthesized CeO_2_NPs, Al_2_O_3_NPs, and CeO_2_/Al_2_O_3_ nanocomposite FT-IR spectra were measured in the 400–4000 cm^−1^ region. CeO_2_NPs’ FT-IR spectrum (Figure 5a) revealed absorption bands at 3400 cm^−1^ (O-H stretching vibration), 1626 cm^−1^ (O-H bending vibration of absorbed water), and 488 cm^−1^ (Ce-O-Ce) stretching vibration and 555 cm^−1^ (formation of Ce-O stretching bond). The obtaining results are consistent with prior findings [53]. The FT-IR spectrum of the Al_2_O_3_NPs is described in Figure 5b.

The broad absorption bands, appeared at 3466 cm^−1^ and 1632 cm^−1^, resulted from stretching and bending O-H vibration of absorbed water, respectively. The stretching vibration of Al-OH bond appeared to correspond another band observed at 1362 cm^−1^. The peaks at 832 cm^−1^ and 586 cm^−1^ correspond to the Al-O bond [54]. The CeO_2_/Al_2_O_3_ nanocomposite spectrum displayed various absorption bands at 3464 cm^−1^ (O-H), 2374 cm^−1^ (O=C=O of the carbon dioxide), and 1630 cm^−1^ (O-H vibration mode of water). The formation of CeO_2_/Al_2_O_3_ nanocomposite was confirmed by the appearance of stretching vibration peaks at 562 and 832 cm^−1^ (Figure 5c). The shift of the peaks in the nanocomposite spectrum to 562 and 832 cm^−1^ indicating the incorporation of CeO_2_ nanoparticles on the surface of Al_2_O_3_ nanoparticles.

**Figure 6:**
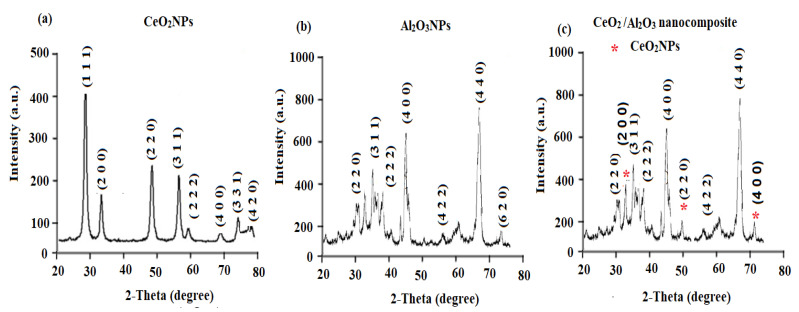
XRD patterns of (**a**) CeO_2_NPs, (**b**) Al_2_O_3_NPs, and (**c**) CeO_2_/Al_2_O_3_ nanocomposite measured in the range of 2θ degree between 20–80°.

The XRD patterns of CeO_2_NPs, Al_2_O_3_NPs, and CeO_2_/Al_2_O_3_ nanocomposite were scanned from 10–80 degrees with a 2θ min-1 scan rate. The XRD pattern of CeO_2_NPs showed different intensity peaks corresponding to crystal planes at 28.41° (1 1 1), 33.62° (2 0 0), 48.38° (2 2 0), 57.74° (3 1 1), 59.03° (2 2 2), 69.37° (4 0 0), 76.69° (3 3 1), and 79.09° (4 2 0) crystal planes (Figure 6a) and these results are matched those previously reported as in JCPDS Card No.-34-0394 [54]. The XRD pattern of CeO_2_NPs revealed the formation of pure crystalline cubic fluorite in shape. The XRD pattern of Al_2_O_3_ nanoparticles showed cubic and symmetric crystals with face-centered lattice (Figure 6b). The obtained 2θ values were found at 32.5° (2 2 0), 35.1° (3 1 1), 38.7° (2 2 2), 46.5° (4 0 0), 62.4° (4 2 2), 67.2° (4 4 0), 78.4° (6 2 0). These values were in agreement with JCPDS Card No. 79-1558 [55]. However, the XRD pattern of CeO_2_/Al_2_O_3_ nanocomposite showed distinctive peaks for CeO_2_NPs at 33.62° (2 0 0), 48.38° (2 2 0), and 69.37° (4 0 0) indicating the formation of CeO_2_/Al_2_O_3_ nanocomposite (Figure 6c). Moreover, the Scherer equation was used to calculate the average size of CeO_2_NPs, Al_2_O_3_NPs, and CeO_2_/Al_2_O_3_ nanocomposite by obeying:

D = 0.9λ/βCosθ(2)

where D, λ, β, and θ represent crystallite size, wavelength, the half-width of the diffraction peak, and the diffraction angle of the highest peak, respectively [56]. The average crystallite size obtained for CeO_2_NPs, Al_2_O_3_NPs, and CeO_2_/Al_2_O_3_ nanocomposite was found to be 17.35 nm, 18.80 nm, and 21.66 nm, respectively.

The dislocation density (δ) is identified as the length of dislocation lines per unit volume of the crystal, which reflects number of defects in the sample and is estimated using the following equation [57].

δ = 1/D^2^ (3)

where the crystallite size is donated by D. The dislocation density of CeO2NPs and Al2O3NPs at room temperature was found to be 6.94 × 10^−3^ and 1.31 × 10^−3^ (nm)^−2^, respectively. The equation [58] was used to calculate the length of Ce-O and Al-O bond.

(4) $$\sqrt{(\frac{a^{2}}{3}+\left(\frac{1}{2}-u{)}^{2}c^{2}\right)}$$

The values in the above equation expressed as ‘’u’’ the potion in the wurtzite shape and can be determined by measuring the displacement of the atom with respect to the next atom along the axis ‘’c’’.

Where u is the positional parameter in the wurtzite structure and is a measure of the amount by which each atom is displaced according to the next along the ‘c’ axis. ‘u’ is given by the equation:

(5) $$u=\frac{a^{2}}{3c^{2}}+0.25\;$$

The Ce-O and Al-O bond lengths were calculated to be 1.869Å and 1.987Å, respectively. The estimated values matched the unit cell of Ce-O and Al-O bond lengths [59,60].

**Figure 7:**
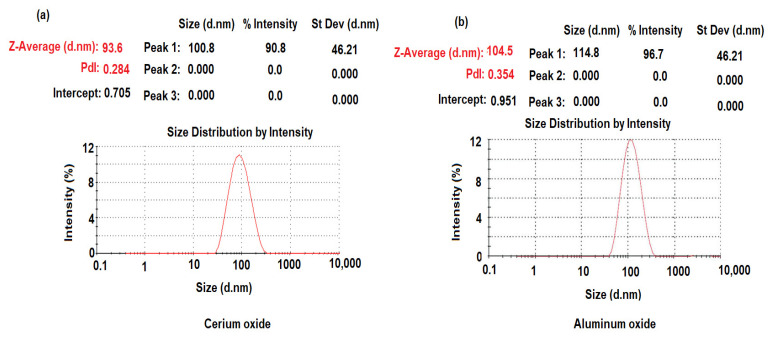
Dynamic light scattering of pre-synthesized (**a**) CeO_2_NPs and (**b**) Al_2_O_3_NPs.

The dynamic light scattering (DLS) method was applied to measure the mean size-average diameter (d.nm) and the size distribution by the intensity of the synthesized CeO_2_NPs and Al_2_O_3_NPs. The particle size distribution of CeO_2_NPs and Al_2_O_3_NPs was measured using a particle size analyzer Zetasizer Ultra (Malvern Panalytical Ltd., Malvern, UK). As demonstrated in Figure 7a,b, the particle size distribution of CeO_2_NPs and Al_2_O_3_NPs was about 93.6 ± 2.4 and 104.5 ± 0.6 nm, respectively.

**Figure 8:**
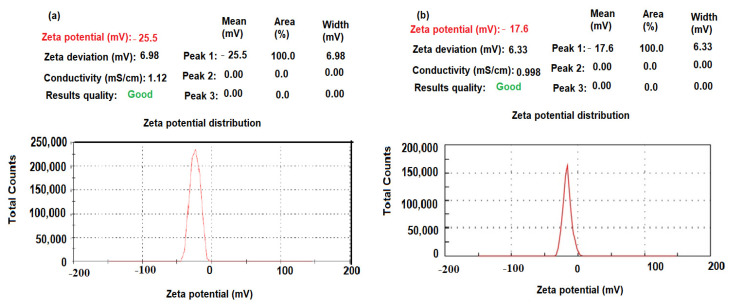
Zeta potential of pre-synthesized (**a**) CeO_2_NPs and (**b**) Al_2_O_3_NPs.

The size distribution profiles of CeO_2_NPs and Al_2_O_3_NPs exhibited one remarkable peak for each with intensities 90.8%, 96.7%, respectively, with a polydispersity index (PdI) 0.284 and 0.354 for CeO_2_NPs and Al_2_O_3_NPs, respectively, suggesting that the synthesized nano metal oxides had a little agglomeration [61]. The zeta potential of pre-synthesized CeO_2_NPs and Al_2_O_3_NPs with negative values of about −25.5 and −17.6 mV indicated a strong negative charge (Figure 8a,b). The negative surface zeta potential of CeO_2_NPs and Al_2_O_3_NPs suggests their reduction in metal oxide nanoparticles.

**Figure 9:**
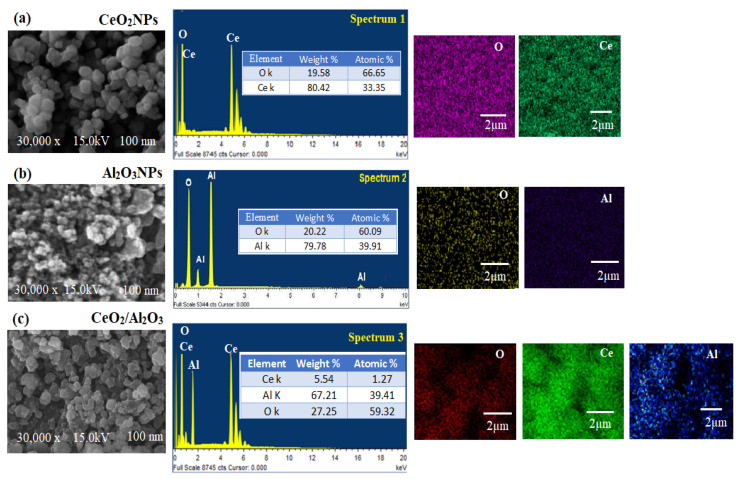
SEM, EDX and elemental mapping of (**a**) CeO_2_NPs, (**b**) Al_2_O_3_NPs, and (**c**) CeO_2_/Al_2_O_3_ nanocomposite.

The surface morphology, and elemental presence in the pre-synthesized CeO_2_NPs, Al_2_O_3_NPs, and CeO_2_/Al_2_O_3_ nanocomposite was visualized by SEM coupled with EDX (Figure 9a–c). The images of SEM showed that most of the synthesized CeO_2_NPs were cubic fluorite in shape (Figure 9a), whereas, the SEM images of the pre-synthesized Al_2_O_3_NPs revealed quasi-spherical shape in (Figure 9b); however, in the synthesized CeO_2_/Al_2_O_3_ nanocomposite, the surface of Al_2_O_3_ was clustered by CeO_2_NPs, the shape was changed to the lattice arrangement of the nanocomposite (Figure 9c). Thus, CeO_2_/Al_2_O_3_ nanocomposite was rounded in shape with an average of size 100 nm. The elemental composition of CeO_2_NPs, Al_2_O_3_NPs, and CeO_2_/Al_2_O_3_ nanocomposite measured by EDX showed the presence of Ce with a weight percentage 80.42% and atomic percentage 33.35% and O with 19.58% and 66.65%, respectively.

The EDX spectrum of Al_2_O_3_NPs confirmed the presence of weight % (79.78% and 20.22%) and atomic % (39.91% and 60.09%) for Al and O, respectively; however, CeO_2_/Al_2_O_3_ nanocomposite spectrum showed the presence of Ce, Al, and O elements with weights of 5.54%, 67.21%, and 27.25%, atomic percentage of 1.27%, 39.41%, and 59.32%, respectively. The atomic arrangement of pre-synthesized CeO_2_NPs, Al_2_O_3_NPs, and CeO_2_/Al_2_O_3_ nanocomposite was evaluated by EDX mapping analysis. Figure 9a also showed the mapping of CeO_2_NPs, where Ce ions are spread over the O, while the mapping images of Al_2_O_3_NPs showed mutual spreading of Al and O (Figure 9b); however, CeO_2_/Al_2_O_3_ nanocomposite mapping spectrum exhibited the content of Al was higher than Ce and O (Figure 9c). Furthermore, the decoration of Ce with Al and O atoms was noticed in the mapping analysis of CeO_2_/Al_2_O_3_ nanocomposite.

### 3.2. Performance Response of the Suggested Sensors

**Figure 10:**
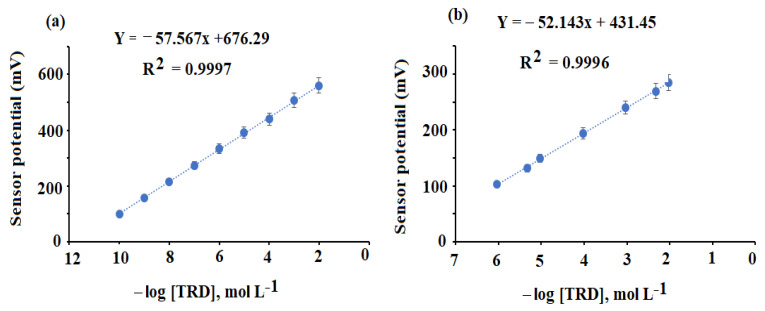
Calibration graphs of the constructed TRD-PM (**a**) and functionalized TRD-PM- CeO_2_/Al_2_O_3_ nanocomposite (**b**).

TRD interacts with PMA to form the TRD-PM complex, which is extremely stable and soluble in THF. THF was used to combine the membrane cocktail to create the conventional TRD-PM and functionalized coated wire TRD-PM-CeO_2_/Al_2_O_3_NPs nanocomposite sensors. The application of (o-NPOE, = 24) with a high dielectric constant increases membrane complex uniform solubility and computability with the polymeric phase of the membrane; it also improves the sensor’s selectivity coefficient by providing a mechanical property for the covered membrane [62]. The potential responses of TRD-PM and TRD-PM-CeO_2_/Al_2_O_3_NPs nanocomposite were described in Table 1. The achieved data indicated that the developed sensors demonstrated Nernstian behavior with slopes of E_mV_ = (52.143 ± 0.5) log [TRD] + 431.45 and E_mV_ = (57.567 ± 0.2) log [TRD] + 676.29 for the above-mentioned sensors, respectively, with linearity ranges of 1.0 × 10^−6^–1.0 × 10^−2^ mol L^−1^ (r^2^ = 0.9996) and 1.0 × 10^−10^–1.0 × 10^−2^ mol L^−1^ (r^2^ = 0.9997 (Figure 10a,b). The addition of CeO_2_/Al_2_O_3_ nanocomposite to the traditional TRD-MP sensor improved to design new functionalized system (TRD-MP-CeO_2_/Al_2_O_3_ nanocomposite) developed sensor’s potential response to a larger linear detection range with increased sensitivity concerning the TRD solution detection. Those data could result from the huge surface area of the additional nanoparticles that enhanced the conductivity of surface of the planned functionalized sensor. Furthermore, the high dielectric permittivity values of CeO_2_NPs (~23) and Al_2_O_3_NPs (~7.8–11.1) at ambient temperature may account for the better detection results achieved with the functionalized sensor [63,64].

The dynamic responsiveness of the created TRD-PM conventional and functionalized TRD-PM-CeO_2_/Al_2_O_3_NPs nanocomposite sensors was examined under ideal experimental environments to identify differences between the time of instant potential and the value of its steady-state (1 mV). The above-mentioned conventional and functionalized sensors had dynamic responses of 60 and 35 s, respectively. The sensor enhanced with metal oxide nanocomposite has a faster response time and more mechanical stability than the standard sensor. The electrical conductivity of the modified sensor towards detection of TRD in the sample is improved by the functionalization of the membrane with metal oxides nanocomposite (high surface area: volume ratio) and their new advanced features. Furthermore, when nanoparticles are utilized as conductive materials in sensing systems, the nanocomposite’s remarkable electrical and capacity features, including significant charge transfer at nanomaterial interfaces, are critical [65].

**Figure 11:**
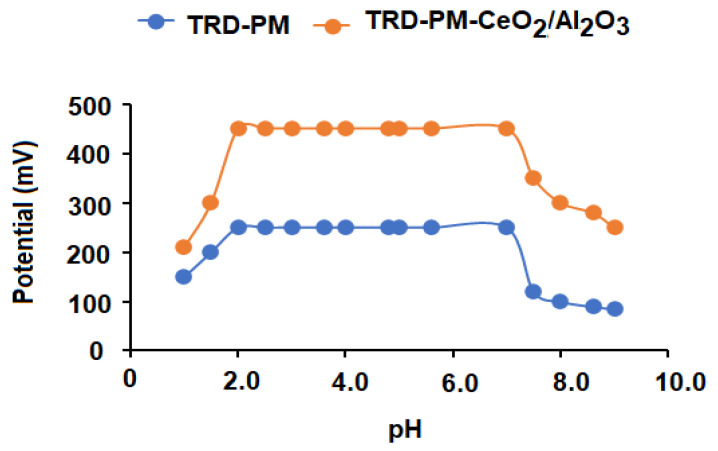
Effect of hydrogen ion concentration (pH) on the potential readings of the conventional and functionalized metal oxide nanocomposite sensors using 1.0 × 10^−4^ mol L^−1^ TRD solution.

The hydrogen ion concentration has a significant impact on the membrane sensor’s potential response. Thus, determining the appropriate pH range where hydrogen ions have no effect on the coated membrane sensor’s potential response is critical. The results revealed that the response of TRD-PM and TRD-PM-CeO_2_/Al_2_O_3_NPs nanocomposite sensors are unaffected in the pH range 2–7, and that can be easily predicted using the developed sensors in this pH range (Figure 11). The protonated ion-pair complex was formed at high [H^+^] in an acidic medium (pH 2), and the sensor potential readings were marginally augmented as a result of low responsiveness to TRD ions; meanwhile, in alkaline medium (pH > 7) where [OH^−^] is high, the potential readings were progressively reduced due to the competition between TRD ions and OH- ions. Consequently, this decreases interactions between the investigated drug ions and sites of ion-pair on the sensor membrane [66].

A separate solution approach [67] was employed to assess the effect of interference of several foreign constituents on the coefficient of selectivity of the developed TRD sensors. The functionalized TRD-PM-CeO_2_/Al_2_O_3_ nanocomposite sensor exhibited high selectivity towards the detection of TRD. The extraordinary physicochemical properties of the produced CeO_2_/Al_2_O_3_NPs, as well as their large interfacial area, improve the conductivity of the modified sensor and therefore its selectivity for TRD ions. Furthermore, the free energy transfer of ions (TRD^+^) created between the active sites in the membrane and the working solution is referred to by the TRD coated membrane selectivity. The evaluated cations, sugars, and amino acids caused no interference. As a result, using the modified TRD sensor for TRD determination provided high selectivity and tolerance (Table 2).

### 3.3. Quantification of TRD in Bulk Form

The TRD drug was determined in its authentic samples using the designed conventional TRD-PM and TRD-PM-CeO_2_/Al_2_O_3_ sensors, and the findings were expressed as 98.80 ± 0.9% and 99.81 ± 0.2%, respectively (Table 3). The use of functionalized TRD-PM-CeO_2_/Al_2_O_3_ sensor containing CeO_2_NPs (~23) and Al_2_O_3_NPs (~7.8–11.1) improved the dynamic detection of the TRD solution.

### 3.4. Validation of the Suggested Method

The guideline of the International Council for Harmonization of Technical Requirements for Pharmaceuticals (ICH) [48] was obeyed to prove the validity and suitability of the designed potentiometric systems for the determination of TRD. The suggested TRD-PM and TRD-PM-CeO_2_/Al_2_O_3_ nanocomposite exserted linear relationships with least square regression equations E_mV_ = (52.143 ± 0.2) log [TRD] + 431.45 (r^2^ = 0.9997) and E_mV_ = (57.25 ± 0.4) log [TRD] + 676.29 (r2 = 0.9998) over the TRD concentration ranges 1.0 × 10^−6^–1.0 × 10^−2^ and 1.0 × 10^−10^–1.0 × 10^−2^ mol L^−1^. Also, the suggested TRD-PM and TRD-PM-CeO_2_/Al_2_O_3_ nanocomposite sensors displayed detection limits of 5.0 × 10^−7^ and 5.0 × 10^−11^ mol L^−1^, respectively.

To study the accuracy of designed sensors, 9 authentic TRD concentrations in the range of 1.0 × 10^−6^–1.0 × 10^−2^ and 1.0 × 10^−10^–1.0 × 10^−2^ mol L^−1^ were used. The accuracy of the suggested potentiometric approach was expressed as mean percentage recoveries of 98.56 ± 0.8% and 99.85 ± 0.2% for TRD-PM and TRD-PM-CeO_2_/Al_2_O_3_ nanocomposite, respectively (Table 4). Intermediate precision experiments were also used to investigate the precision of the suggested functionalized potentiometric TRD-PM-CeO_2_/Al_2_O_3_ nanocomposite system. For the two, the recorded data were found as a percentage relative standard deviation (% RSD) of 0.2% and 0.4%, for intra-day and inter-day assays, respectively (Table 5).

The potentiometric system’s robustness was tested by altering the pH of working solutions to 7 ± 0.5, which was a minor change in the procedure parameter. The obtained percentage recoveries for the standard and functionalized TRD coated wire sensors were 98.35 ± 0.5% and 99.66 ± 0.3%, respectively (Table 1). To prove the ruggedness of the proposed technique, TRD samples were analyzed using a different pH-meter (Metrohm model-744) in another laboratory and by a different analyst. For the above designed TRD-PM and TRD-PM-CeO_2_/Al_2_O_3_ nanocomposite sensors, the mean percentage recoveries were found to be 98.83 ± 0.7 and 99.72 ± 0.4 percent, respectively (Table 1). The results of technique validation were in suitable accordance with those obtained using the suggested system, with no notable variations.

### 3.5. Estimation of TRD in Tramadol hydrochloride^®^ Capsules

The examined TRD was determined utilizing the designed TRD-PM and TRD-PM-CeO_2_/Al_2_O_3_ nanocomposite in its marketed capsules tramadol hydrochloride^®^ (50 mg/ capsule). The % recoveries of TRD were obtained by the regression equations using the potential readings of the working solutions 1.0 × 10^−6^–1.0 × 10^−2^ and 1.0 × 10^−10^–1.0 × 10^−2^ mol L^−1^. For the above-mentioned sensors, the recorded results were 98.77 ± 0.8% and 99.63 ± 0.5%, respectively. The obtained findings were compared to the approach provided by Shawish et al. [68] using the Student’s *t*-test and F-test [69] and revealed that the developed sensor had outstanding sensitivity and selectivity for the determination of TRD (Table 6).

The dielectric constant is an important criterion for determining a material’s ability to hold charges [70]. Electronics and sensors frequently use metal oxides with a high dielectric constant. They allow for the exertion of an electrostatic field and hence the storage of charges because they do not allow for the flow of charges through them [71]. The electrical, optical, and conductive capabilities of the functionalized sensor might all be improved by combining metal oxide nanoparticles with a polymeric medium in nanocomposites. Changes in the shape and size of the particles have a big impact on these qualities. As previously addressed, nanoparticles can act as a conductive connection between the polymeric chains, resulting in an increase in the composites’ electrical conductance [72,73]. The efficiency of the designed functionalized TRD-PM-CeO_2_/Al_2_O_3_ nanocomposite sensor was compared to that of previously constructed sensors [69,73,74] (Table 7). The modified sensor had a higher sensitivity than the published sensors for detecting TRD, with a detection range of 1.0 × 10^−10^–1.0 × 10^−2^ and a LOD of 5.0 × 10^−11^ mol L^−1^. The most significant component in developing ultrasensitive sensors with required features is the choice of nanostructured materials and sensor design method. The surface-to-volume ratio, which is a critical component in enhancing contact reactions on the overall electrical conductivity of nanomaterials, is determined by the shape and size of the nanoparticles utilized. Thus, due to the great chemical stability of these nanomaterials, the nanoscale morphology will affect not only the sensitivity of the sensor but also the dynamic responsiveness and long-term stability of the sensor. The molecular structure and polymeric media, such as crystallinity and long-chain polymer, may influence the electrical conductivity of metal oxide nanocomposite-fabricated sensors [75].

## 4. Conclusions

The current study describes a simple and ultrasensitive functionalized TRD-PM-CeO_2_/Al_2_O_3_ nanocomposite potentiometric sensor for determining TRD in authentic powder and commercial formulations that was successfully built. The modified sensor had a large surface area-to-volume ratio, which gave it excellent sensitivity in the detection of TRD with linear relationships in the concentration ranges 1.0 × 10–6–1.0 × 10–2 and 1.0 × 10–10–1.0 × 10–2 mol L^−1^, and low detection limits of 5.0 × 10–6 and 5.0 × 10–11 mol L^−1^ for the conventional and functionalized sensors, respectively, with least square regression equations EmV = (52.143 ± 0.4) log [TRD] + 431.45 and EmV = (57.567 ± 0.2) log [TRD] +676.29 for the above described TRD sensors, respectively. The results of the proposed method were statistically assessed and compared to those of sensors that had previously been reported. The modified TRD-PM-CeO_2_/Al_2_O_3_ nanocomposite was shown to have a substantially greater potential response than the standard kind. Furthermore, coating the sensor’s surface with a modified layer of metal oxide nanocomposite polymeric membrane improves the sensor’s electroconductivity and quantification of the tested TRD in capsules, with a mean percentage recovery of 99.63 ± 0.5 percent for the TRD-PM-CeO_2_/Al_2_O_3_ nanocomposite sensor, indicating high sensitivity and selectivity. As a result, the use of metal oxide nanocomposite in the construction of polymeric sensors opens up a promising avenue for the development of unique modified potentiometric sensors.

## Figures and Tables

**Table 1:** Performance response characteristics of designed conventional (TRD-PM) and functionalized TRD-PM-CeO_2_/Al_2_O_3_ nanocomposite sensors.

Parameter	TRD-PM Coated Wire Sensor	Functionalized TRD-PM-CeO_2_/Al_2_O_3_ Coated Wire Sensor
Linearity (mol L^−1^)	1.0 × 10^−6^–1.0 × 10^−2^	1.0 × 10^−10^–1.0 × 10^−2^
Least square equation	E_mV_ = (52.143) log [TRD] + 431.45	E_mV_ = (57.567 ± 0.2) log [TRD] + 676.29
Correlation coefficient (r)	0.9997	0.9998
Slope	52.143	57.567
Lower limit of detection	5.0 × 10^−7^	5.0 × 10^−11^
pH	2–7	2–7
Optimum temperature °C	25	25
Response time (s)	60	35
Life time (day)	20	35
Accuracy	98.56 ± 0.8	99.85 ± 0.2
Robustness	98.36 ± 0.5	99.66 ± 0.3
Ruggedness	98.83 ± 0.7	99.72 ± 0.4

**Table 2:** Outcomes of tolerable values (K^pot^_TRD_^+^) of some interferents determined by the suggested TRD-PM and functionalized TRD-PM-CeO_2_/Al_2_O_3_ nanocomposite coated wire sensors using separate solution approach.

Foreign Substance	TRD-PM Coated Wire Sensor	Functionalized TRD-PM-CeO_2_/Al_2_O_3_ Coated Wire Sensor
Na^+^	2.38 × 10^−4^	1.12 × 10^−6^
K^+^	1.56 × 10^−3^	1.89 × 10^−5^
Ag^+^	5.07 × 10^−4^	4.25 × 10^−5^
Mg^2+^	2.16 × 10^−6^	3.63 × 10^−7^
Ca^2+^	5.70 × 10^−4^	7.51 × 10^−6^
Zn^2+^	1.84 × 10^−3^	1.02 × 10^−8^
Lactose	4.47 × 10^−3^	6.35 × 10^−4^
Fructose	3.86 × 10^−3^	1.89 × 10^−6^
Starch	2.74 × 10^−4^	5.89 × 10^−5^
Histidine	5.49 × 10^−3^	3.74 × 10^−4^
Glycine	1.29 × 10^−5^	1.11 × 10^−6^
Lysin	2.34 × 10^−3^	2.39 × 10^−7^
Tryptophan	1.23 × 10^−3^	1.57 × 10^−6^

**Table 3:** The obtained results of the determination of TRD in its authentic samples using conventional and functionalized TRD-PM and TRD-PM-CeO_2_/Al_2_O_3_ nanocomposite coated wire sensors.

	TRD-PM Coated Wire Sensor		Functionalized TRD-PM-CeO_2_/Al_2_O_3_ Coated Wire Sensor	
	Taken Sample -log [TRD] mol L^−1^	% Recovery	Taken Sample -log [TRD] mol L^−1^	% Recovery
Statistical analysis	6	99.7	10	99.9
	5.3	98.9	9	100
	5	99.6	8	99.8
	4	98.8	6	99.6
	3.3	98.8	5	99.5
	3	97.3	4	99.9
	2.3	97.8	3	99.8
	2	99.5	2	100
Mean ± SD	98.80 ± 0.9		99.81 ± 0.2	
n	8		8	
Variance	0.81		0.04	
%RED	0.91		0.2	
%Error *	0.32		0.07	

* % Error = %RSD/$\sqrt{n}$.

**Table 4:** Accuracy results of the analysis of TRD samples using conventional and functionalized TRD-PM and TRD-PM-CeO_2_/Al_2_O_3_ nanocomposite coated wire sensors.

	TRD-PM Coated Wire Sensor		Functionalized TRD-PM-CeO_2_/Al_2_O_3_ Coated Wire Sensor	
	Taken Sample -log [TRD] mol L^−1^	% Recovery	Taken Sample -log [TRD] mol L^−1^	% Recovery
Statistical analysis	6	98.9	10	100
	5.3	98.5	9	99.8
	5	99.6	8	99.7
	4.3	97.3	6	100
	4	97.6	5	99.6
	3.3	98.7	4	99.8
	3	99.1	3	99.7
	2.3	98.5	2.3	99.9
	2	99.3	2	100
Mean ± SD	98.56 ± 0.8		99.85 ± 0.2	
n	9		9	
Variance	0.64		0.04	
%RED	0.81		0.2	
%Error *	0.28		0.07	

* % Error = %RSD/$\sqrt{n}$.

**Table 5:** Precision (inter-day and intra-day assays) results for the determination of TRD samples by using conventional and functionalized TRD-PM and TRD-PM-CeO_2_/Al_2_O_3_ nanocomposite coated wire sensors.

	Functionalized TRD-PM-CeO_2_/Al_2_O_3_ Coated Wire Sensor					
	Intra-Day Assay			Inter-Day Assay		
	Sample	Found	%	Sample	Found	%
Statistical analysis	-log [TRD]	-log [TRD]	Recovery	-log [TRD]	-log [TRD]	Recovery
	mol L^−1^	mol L^−1^		mol L^−1^	mol L^−1^	
	10	9.99	99.9	10	10.02	100.2
	8	7.97	99.6	8	7.99	99.9
	2	2	100	2	1.99	99.5
Mean ± SD	99.83 ± 0.2			99.86 ± 0.4		
n	3			3		
Variance	0.04			0.16		
%RED	0.2			0.4		
%Error *	0.12			0.23		

* % Error = %RSD/$\sqrt{n}$.

**Table 6:** The obtained results of the determination of TRD in its commercial capsules (50 mg/capsule) using conventional and functionalized TRD-PM and TRD-PM-CeO_2_/Al_2_O_3_ nanocomposite coated wire sensors.

	TRD-PM Coated Wire Sensor		Functionalized TRD-PM-CeO_2_/Al_2_O_3_ Coated Wire Sensor		
	Sample -log [TRD]	Recovery	Sample -log [TRD]	% Recovery	
Statistical analysis	mol L^−1^		mol L^−1^		Reported Method [68]
	6	98.9	10	99.6	
	5.3	99.8	8	99.7	
	5	98.7	6	99.9	
	4	97.9	4	98.6	
	3	97.8	3	99.9	
	2	99.5	2	100	
Mean ± SD	98.77 ± 0.8		99.63 ± 0.5		
n	6		6		99.25 ± 0.6
Variance	0.64		0.25		6
%RED	0.81		0.5		0.36
%Error *	0.33		0.2		0.6
*t*-test	1.176 (2.228) *		1.216 (2.228) *		0.24
F-test	1.77 (5.05) *		1.44 (5.05) *		

* Tabulated values of Student’s *t*-test and F-test at *p* < 0.05 [69].

**Table 7:** Comparative results between the suggested conventional and functionalized TRD-PM and TRD-PM-CeO_2_/Al_2_O_3_ nanocomposite coated wire sensors.

No.	Ion-Pair Complex	Linear Concentration Range (mol L^−1^)	LOD (mol L^−1^)	Reference
1.	TRD-Phosphomolybdic acid	2.0 × 10^−6^–1.0 × 10^−1^	1.3 × 10^−5^	[73]
2.	TRD-Phosphotunegstic acid	9.0 × 10^−6^–1.0 × 10^−1^	6.2 × 10^−6^	[69]
3.	TRD-Silico-tungstic acid	2.0 × 10^−6^–1.0 × 10^−1^	4.0 × 10^−6^	[74]
4.	TRD-Phosphomolybdic acid	1.0 × 10^−10^–1.0 × 10^−2^	5.0 × 10^−11^	Current study

## Data Availability

The collected data from the current study was included in the text.
